# Natural products targeting human lactate dehydrogenases for cancer therapy: A mini review

**DOI:** 10.3389/fchem.2022.1013670

**Published:** 2022-09-29

**Authors:** Huankai Yao, Feng Yang, Yan Li

**Affiliations:** ^1^ Department of Microbial and Biochemical Pharmacy, School of Pharmacy, Jiangsu Key Laboratory of New Drug Research and Clinical Pharmacy, Xuzhou Medical University, Xuzhou, Jiangsu, China; ^2^ School of Stomatology, Xuzhou Medical University, Xuzhou, Jiangsu, China

**Keywords:** natural products, lactate dehydrogenase, cancer metabolism, Warburg effect, inhibitors

## Abstract

Reprogramming cancer metabolism has become the hallmark of cancer progression. As the key enzyme catalyzing the conversion of pyruvate to lactate in aerobic glycolysis of cancer cells, human lactate dehydrogenase (LDH) has been a promising target in the discovery of anticancer agents. Natural products are important sources of new drugs. Up to now, some natural compounds have been reported with the activity to target LDH. To give more information on the development of LDH inhibitors and application of natural products, herein, we reviewed the natural compounds with inhibition of LDH from diverse structures and discussed the future direction of the discovery of natural LDH inhibitors for cancer therapy.

## Introduction

**FIGURE 1:**
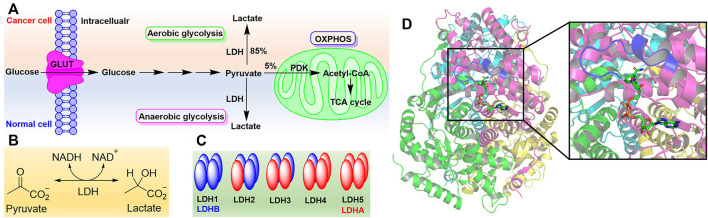
The Warburg effect and lactate dehydrogenase. **(A)** The Warburg effect. **(B)** Reaction catalyzed by LDH. **(C)** Isoforms of LDH. **(D)** Structure of LDH5 with the binding mode of NADH (PDB code 1I10). Four monomers (LDHA) were rendered with magenta, cyan, green, and yellow, respectively. Active site loop was highlighted in blue. NADH was shown as sticks.

In recent years, metabolic reprogramming has attracted more attention regarding cancer progression, as cancer cells have a distinct metabolism compared to normal cells (Hay, 2016). In normal cells, the glucose is transported into cytosol by glucose transporters (GLUT) and converted into pyruvate under the catalysis of a series of enzymes. The latter will enter into the mitochondria and degrade to form acetyl-CoA catalyzed by pyruvate dehydrogenase complex (PDC) to generate more ATP if there is enough of an oxygen source, which is called oxidative phosphorylation (OXPHOS). Under hypoxic conditions, the pyruvate will be converted to lactate under the catalysis of lactate dehydrogenase (LDH) following the oxidation of NADH to NAD^+^ as anaerobic glycolysis (Cairns et al., 2011). However, in cancer cells, the high amounts of glucose are taken up to produce more ATP and meet the requirements of cellular proliferation. Meanwhile, most lactate is produced when enough oxygen is available (Figure 1A). This phenomenon is usually termed as aerobic glycolysis or the Warburg effect (Vander Heiden et al., 2009).

Of all the enzymes in cancer metabolism, LDH is a key node of aerobic glycolysis since this pathway affords the conversion of about 85% pyruvate to lactate (Figure 1B) (Zhang et al., 2015). In many cancer cells, such as gastric cancer (Ping et al., 2018), colorectal cancer (Wang et al., 2015), lung cancer (Kayser et al., 2010), liver cancer (Faloppi et al., 2016), breast cancer (Arundhathi et al., 2021), brain cancer (Valvona et al., 2016), bladder cancer (Burns et al., 2021) and so on, it was found that LDH has been expressed excessively. LDH is closely associated with the diagnosis, treatment, and prognosis of cancer patients as it is involved in all stages of cancer progression. Targeting LDH not only inhibits the proliferation, tumorigenesis, and progression, but also suppresses the invasion, metastasis, and angiogenesis of cancer (Feng et al., 2018). Therefore, inhibiting LDH to target cancer metabolism is a potential therapeutic approach to discover anticancer agents (Zhang et al., 2018; Stine et al., 2022).

As the NADH-dependent enzyme, lactate dehydrogenase is a tetramer composed of two major subunits, LDHA (also known as LDH-M) and LDHB (also known as LDH-H), which are encoded by *LDHA* and *LDHB* genes, respectively (Markert et al., 1975). Therefore, according to the number of different subunits, there are five isoforms of LDH, named LDH1-LDH5 (Figure 1C) (Dawson et al., 1964). The metabolic characteristics of LDH isoforms are determined by the composition of subunits. LDHA preferentially reduces pyruvate to lactate while LDHB kinetically favors the conversion of lactate to pyruvate (Echigoya et al., 2009; Augoff et al., 2015). Hence, LDHA or LDH5 often attracts medicinal chemists’ attention as the target to regulate cancer metabolism (Rani and Kumar, 2017; Zhang et al., 2018). In addition, there some other isoforms of LDH including LDHC, LDHD, and LDHBx. LDHC, also known as LDHX, is testes-specific (Blanco and Zinkham, 1963; Burkhart et al., 1982). Until now, the role of LDHD has been less understood, and it is reported that LDHD is responsible for the metabolism of D-lactate *in vivo* (Monroe et al., 2019). LDHBx localized in the peroxisome and is generated by translational readthrough (Schueren et al., 2014).

Crystal structure analysis of LDH has revealed there are 331 amino acid residues in LDHA and two major binding sites to catalyze the conversion of pyruvate to lactate (Figure 1D) (Read et al., 2001). One is the mixed α/β substrate binding site including residues 163–247 and 267–331, which is enclosed by the active site loop (residues 99–110) away from solvent (Woodford et al., 2020). The residue Arg105 in the active site loop is essential to stabilizing the transition state in the hydride-transfer reaction *via* a hydrogen bond with carbonyl of pyruvate (Swiderek et al., 2015). Another is the NADH binding site comprising a central, 6-stranded parallel β-sheet flanked by three helices on each side, which contains a Rossmann-type fold formed by residues 20–162 and 248–266. And NADH binds in a groove at the end of central β-sheet through the residues His 195, Asp168, Arg171, and Thr246, which contribute to the conversion of pyruvate to lactate in the substrate binding site (Read et al., 2001). In addition, the first 20 residues of LDHA at the N-terminus form an unstructured region to interact with the C-terminus of another monomer and give the oligomers (Adams et al., 1970).

**FIGURE 2:**
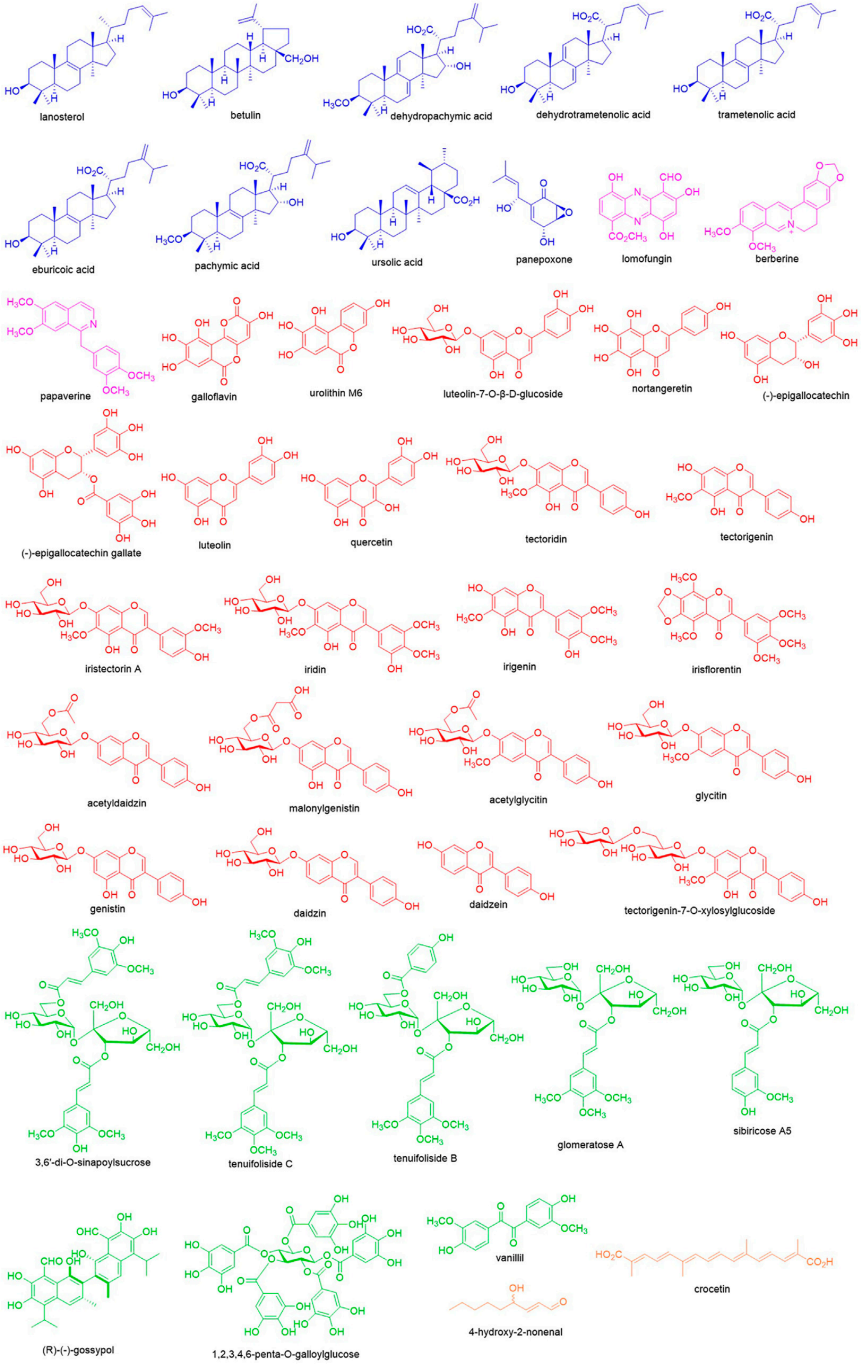
Natural products with potential inhibition of LDH.

In the discovery of new drugs, natural products play a pivotal role (Newman and Cragg, 2020). To find novel LDH inhibitors, some natural compounds have been explored (Figure 2) that showed structural and pharmacological diversity. Herein we summarize these compounds to give insights into the future discovery of LDH inhibitors.

## Natural compounds as lactate dehydrogenase inhibitors

### Terpenoids

Terpenoids are a class of hydrocarbon compounds derived from isoprenoids and classified based on the number of isoprenoid moieties. In the screening of LDH inhibitors, some terpenoids showed significant potential. Panepoxone, a monoterpenoid identified from *Lentinus strigellus*, reduced oxygen consumption, lactate production, and ATP synthesis in breast cancer cells *via* inhibiting LDHA (Arora et al., 2015). Ursolic acid is a ursane-type triterpenoid found in many plants and offers some pharmacological effects. Recently, it was found that ursolic acid at 500 μM inhibited LDHA with the inhibition rate of 30.5% ± 6.7% by electrophoretically mediating microanalysis (Li t al., 2021). Using the ultrafiltration-high speed countercurrent chromatography technique, dehydropachymic acid, pachymic acid, dehydrotrametenolic acid, trametenolic acid, and eburicoic acid from *Poria cocos* were identified as LDH inhibitors as well as lanosterol together with betulin from *Inonotus Obliquus* (Li et al., 2017a; Wang et al., 2021), and most of those are rich in higher fungus such as lanostane-type triterpenoids.

### Alkaloids

Alkaloids are natural compounds containing nitrogen atoms and the secondary metabolites synthesized from amino acids. The first alkaloid as an LDHA inhibitor is berberine. As the major isoquinoline-type alkaloid in *Coptis chinensis*, berberine offers the significant inhibitory constant of 12.6 μM and dissociation constant of 3.1 μM while interacting with LDHA (Kapp and Whiteley, 1991). Further investigation has revealed berberine suppressed progression of pancreatic adenocarcinoma through functionally inhibiting LDHA (Cheng et al., 2021). Additionally, through the visual screening from the NCI Diversity Set, the microbial secondary metabolite lomofungin was selected as an LDHA inhibitor, and enzyme assay has disclosed lomofungin inhibited LDHA with the IC_100_ of 202 μM (Manerba et al., 2012). As an isoquinoline-type alkaloid, papaverine is also reported for its inhibition of LDHA with an inhibitory constant of 196.4 μM and dissociation constant of 52 μM (Kapp & Whiteley, 1991).

### Flavonoids

Flavonoids are the derivatives of chromones and contribute largely to the discovery of natural LDH inhibitors. As the flavanol analogue, galloflavin was found to inhibit LDHA by virtual screening and was validated with the IC_100_ of 201 μM. It was also observed that galloflavin inhibited ATP production in hepatocarcinoma PLC/PRF/5 cells as well as cell growth and respiration (Manerba et al., 2012). Galloflavin can occupy the NADH binding site of LDHA to prevent its binding to single stranded DNA and repress the proliferation of human colorectal cancer SW620 cells (Fiume et al., 2013). Meanwhile, galloflavin inhibited growth of various breast cancer cells *via* suppressing the proliferation and inducing oxidative stress resulting from blocking ATP production and glycolysis (Farabegoli et al., 2012). In Burkitt lymphoma cells, inhibiting LDHA by galloflavin caused MYC down-regulation, which is the most important survival signal (Vettraino et al., 2013). Similarly, urolithin M6, the galloflavin mimetic identified from gut microbiota metabolites, inhibited purified human LDHA with the IC_50_ of 77 ± 10 μM as well as lactate production in Raji cells (IC_50_ of 36 ± 3 μM) and cell growth (IC_50_ of 25 ± 2 μM) (Rupiani et al., 2016). As the major flavanol in green tea, (-)-epigallocatechin gallate inhibits LDHA in MIA PaCa-2 pancreatic cancer cells and confers the anti-cancer activity by disrupting the cellular metabolic network (Lu et al., 2015). And its precursor, (-)-epigallocatechin, as an LDHA inhibitor could significantly inhibit breast cancer growth and induce apoptosis (Wang et al., 2013). In addition, luteolin-7-O-β-D-glucoside was reported to inhibit human LDH5 with the IC_50_ of 139.2 ± 3.1 μM (Bader et al., 2015). And nortangeretin was found to inhibit LDHA with the IC_100_ of 270 μM (Manerba et al., 2012). In the screening of phytochemicals, many flavonoids have been indicated with the potential to inhibit LDH such as quercetin, luteolin, tectoridin, iristectorin A, iridin, tectorigenin, irigenin, irisflorentin, acetyldaidzin, malonylgenistin, daidzin, glycitin, genistin, acetylglycitin, daidzein, and tectorigenin-7-O-xylosylglucoside (Li et al., 2016; Tang et al., 2016; Li et al., 2021).

### Polyphenols

In addition to flavonoids, there are other polyphenols with inhibitory effects on LDH. (R)-(-)-gossypol in cotton processes potent LDH inhibition. However, due to the high toxicity in the heart, kidney, and muscle, its application is hampered and it is hard to further develop it as an anticancer agent (Gomez et al., 1997). 3,6′-Di-O-sinapoylsucrose is a dimer of phenylpropionic acid glucosides obtained from *Polygala flavescens* ssp. *flavescens*. It can inhibit human LDH5 with the IC_50_ of 90.4 ± 4.4 μM through binding to the NADH binding site (De Leo et al., 2017). In addition to 3,6′-di-O-sinapoylsucrose, sibiricose A5, glomeratose A, tenuifoliside B, and tenuifoliside C were also screened as LDH inhibitors from Polygala tenuifolia (Li et al., 2017b). As the dimer of vanillin, vanillil inhibited LDHA with the IC_100_ of 205 μM as well (Manerba et al., 2012). 1,2,3,4,6-Penta-O-galloylglucose is a tannin occurring in Galla Chinensis, which could competitively bind to the NADH binding site of LDHA and inhibit its activity with IC_50_ of 27.32 nM. It was also observed in human breast cancer MDA-231 cells that this compound could block lactate production (IC_50_ = 97.81 μM) and cell division (IC_50_ = 1.2 μM) (Deiab et al., 2015).

### Fatty acids

4-Hydroxy-2-nonenal is an α,β-unsaturated aldehyde, which is generated from the oxidation of n-6 linoleic acid and arachidonic acid. And enzyme activity assay uncovered that it inhibited LDH *via* covalent binding and reduced NADH formation (Ramanathan et al., 2014). Crocetin is a carotenoid found in medicinal plant saffron; its sodium salt could inhibit human LDH5 with the IC_50_ of 54.9 ± 4.7 μM and the proliferation of glycolytic cancer cell lines including human lung cancer A549 cells (IC_50_ = 114.0 ± 8.0 μM) and human cervical cancer HeLa cells (IC_50_ = 113.0 ± 11.1 μM) (Granchi et al., 2017).

## Conclusion and perspective

As more attention is paid to metabolism reprogramming of cancer cells, the importance of the discovery of LDH inhibitors has been raised again. Natural products provide rich resources to find new LDH inhibitors. However, the present status is despondent and some challenges have to be encountered. The first is the work screening natural LDH inhibitors is poor, though the number of natural compounds is large, which may be overcome as more people pursue this avenue and the mature assay methods are employed. Meanwhile, the crystal structure of LDH has been established, which could give more insights into the affinity of natural compounds. The second is weak potency of natural products with a poor drug-like property, which should be improved by medicinal chemists through chemical synthesis. The last is the pharmacological evaluation and exploration of these natural compounds are insufficient and cannot provide sound evidence, especially *in vivo*, which may result from the small amounts obtained from nature. These compounds need to be enriched *via* both synthesis and isolation. In the future, the involvement of pharmacologists together with natural product chemists in the work will enhance those investigations. Collectively, the discovery of LDH inhibitors from natural products is still an attractive approach for cancer therapy, which should integrate multiple disciplines including natural products chemistry, medicinal chemistry, and pharmacology.
